# Minimally Invasive Ceramic Laminate Veneers for Maxillary Anterior Esthetic Rehabilitation: A 12+ Years Follow‐Up

**DOI:** 10.1111/jerd.70001

**Published:** 2025-07-13

**Authors:** José Maurício dos Santos Nunes Reis, Taisa Nogueira Pansani, Marcelo Antonialli Del'Acqua, Filipe de Oliveira Abi‐Rached

**Affiliations:** ^1^ Department of Dental Materials and Prosthodontics São Paulo State University (UNESP), Araraquara School of Dentistry Araraquara Brazil; ^2^ Coordinator of the Advanced Training Course in Fixed Prosthetics São Paulo Association of Dental Surgeons (APCD) Araraquara Brazil

**Keywords:** ceramic veneers, long‐term follow‐up, minimally invasive dentistry, tooth preparation

## Abstract

**Objective:**

This case report describes an esthetic rehabilitation focused on closing diastemas and improving tooth morphology to restore smile harmony using thin porcelain laminates. The advantages, limitations, and characteristics of feldspathic ceramics in laminate fabrication are discussed, highlighting their importance in minimally invasive restorative dentistry.

**Clinical Considerations:**

The growing demand for conservative treatments with excellent esthetics has led to an increased use of laminates in different materials and thicknesses. Feldspathic porcelain is highly valued for its exceptional optical properties and ability to produce outstanding esthetic outcomes. A comprehensive clinical assessment, including anamnesis, examination, and mock‐up planning, was conducted before fabricating ceramic laminates for all the maxillary anterior teeth. Tooth preparations were minimally invasive and enamel‐limited, optimizing the adhesive bonding between glass–ceramic material and tooth substrate. This approach ensured a predictable and long‐lasting outcome.

**Conclusions:**

This case report highlights the significance of meticulous planning, appropriate material and technique selection, and precise execution in achieving a durable, functional, and highly esthetic rehabilitation. After 12+ years of follow‐up, the patient demonstrated excellent clinical outcomes and high satisfaction with the treatment, reinforcing the long‐term effectiveness of thin feldspathic laminates in conservative dental restorations.

## 1 Introduction

With advancements in dental materials and techniques, the pursuit of minimally invasive procedures with high esthetic outcomes has led to a significant increase in the indication of ceramic laminates in various thicknesses [1]. The precise indication, predictability, and success of ceramic laminates are closely linked to the evolution of materials and several technical factors, including enamel and/or dentin acid etching [2], the use of resin cement [3, 4, 5], surface treatment of ceramics, and the selection of strong yet esthetic materials [6, 7, 8, 9]. Additionally, a better understanding of dental preparation plays a crucial role in treatment outcomes.

For successful rehabilitation with ceramic laminates, adequate and precise tooth preparation is essential to ensure proper seating of the restoration, particularly in the cervical region, where it contributes to optimal esthetics and marginal adaptation [10, 11], respecting both restorative and periodontal aspects. However, excessive removal of dental structure can compromise the bond strength between the tooth substrate and the restorative material. Greater dentin exposure poses challenges for adhesion, as bonding to dentin is more complex than to enamel [12, 13]. However, it has been demonstrated and is well described in the literature that performing the Immediate Dentin Sealing (IDS) technique significantly improves the adhesion and fracture resistance of veneers, particularly when more than 50% of the tooth preparation involves dentin exposure [14, 15]. Dentin is composed of approximately 70% mineral phase, 20% protein, and 10% water (by mass), making its adhesion process more susceptible to degradation of the hybrid layer and failure at the adhesive interface. These failures occur due to hydrolytic or enzymatic proteolytic degradation mediated by matrix metalloproteinases present in the tissue [12, 16].

Through contemporary concepts of minimally invasive dentistry, it is possible to fabricate thin conservative ceramic laminates, commonly referred to as “contact lenses,” with thicknesses less than 0.5 mm. These laminates can be cemented onto the tooth surface with minimal dental preparation, allowing modifications in tooth position, shape, size, and color [17, 18, 19, 20, 21, 22, 23, 24], as well as diastema closures [23, 25]. The ceramics recommended for fabricating these laminates, which offer superior esthetic outcomes and excellent optical properties, are those with a high vitreous phase content, as well as hybrid ceramics. In addition to their remarkable esthetic performance, these materials can be conditioned with hydrofluoric acid, enhancing their adhesive properties [5, 8, 11, 26], an essential aspect to the final mechanical resistance and long‐term success of restored teeth.

The most suitable ceramics for this purpose include feldspathic porcelain, leucite‐reinforced, and lithium disilicate glass–ceramics, lithium silicate reinforced with zirconia, and hybrid ceramics such as Vita ENAMIC [27, 28, 29, 30, 31, 32, 33, 34, 35, 36, 37, 38]. The latter consists of an 86% inorganic ceramic matrix and a 14% organic polymeric structure. The aforementioned materials, characterized by a high proportion of vitreous matrix, not only provide outstanding esthetic results but also exhibit excellent adhesion to resin cements after undergoing hydrofluoric acid treatment (material‐dependent application time), followed by silane application [11, 39].

Among the ceramics with a high vitreous matrix content, feldspathic ceramics fabricated on a refractory die enable a reliable and effective procedure for the production of restorations that closely replicate the shape, color, and esthetic effects of natural teeth through a biomimetic stratification process [40, 41]. This procedure ensures adequate marginal fit, an optimal emergence profile, enhanced incisal translucency, and long‐term survival with a low failure rate [42, 43], making it particularly advantageous for thin laminate veneers [44].

This case report aims to present a step‐by‐step esthetic treatment for improving tooth shape and size, and closing diastemas in maxillary anterior teeth using minimally invasive preparations and thin feldspathic laminates, respecting both restorative and periodontal principles [45]. The clinical outcomes observed after over 12 years of follow‐up are presented.

## 2 Clinical Reports

**FIGURE 1:**
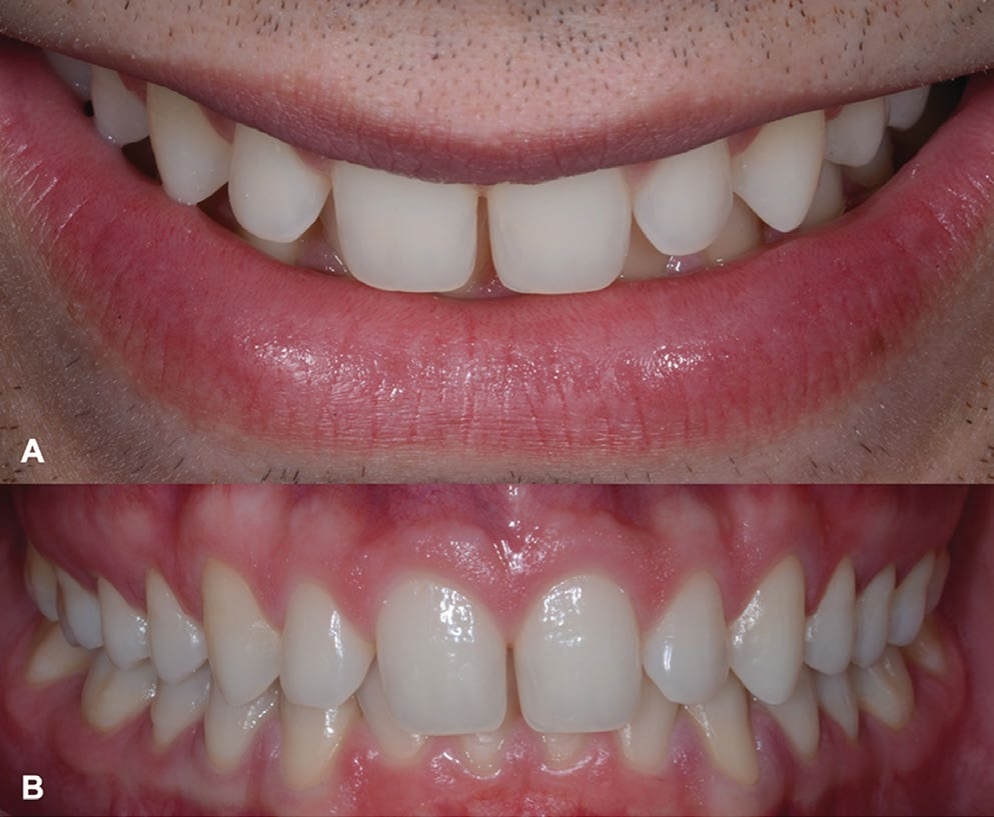
Initial condition showing the youthful appearance of the smile. (A) Frontal view of the smile. (B) Intraoral frontal view.

A 23‐year‐old male patient sought dental care due to dissatisfaction with the esthetics of his smile. His primary concerns included the shape and size of his maxillary anterior teeth and the presence of diastemas. A comprehensive anamnesis and intraoral examination revealed well‐aligned teeth; however, his maxillary anterior teeth exhibited short clinical crowns with a rounded contour and noticeable diastemas, resulting in a youthful appearance of the smile (Figure 1). A minimally invasive rehabilitative approach using feldspathic laminates was proposed to enhance esthetics and close the diastemas. Following informed consent, the patient agreed to treatment, photographic records, and scientific publication of his case.

**FIGURE 2:**
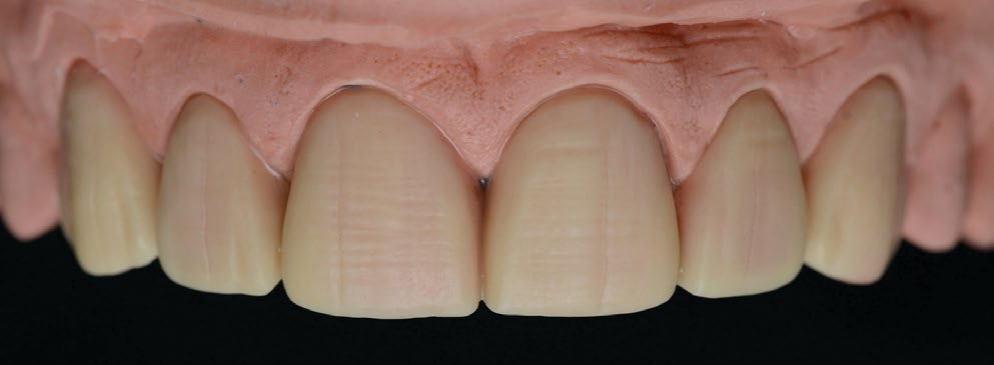
Additive diagnostic waxing.

**FIGURE 3:**
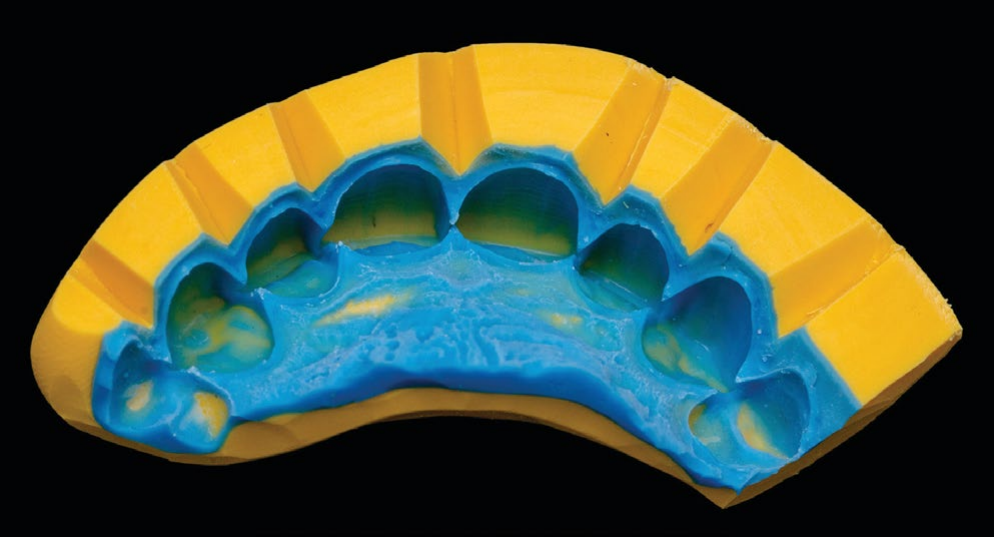
PVS silicone index guide.

A preliminary maxillary impression was taken using condensation silicone (Speedex, Vigodent‐Coltene, Rio de Janeiro, RJ, Brazil) to obtain a study cast with type IV dental stone (Durone, Dentsply, Petrópolis, RJ, Brazil). This cast was used to perform an additive diagnostic wax‐up from canine to canine (Figure 2). Therefore, an index guide (Figure 3) was fabricated with polyvinyl siloxane (PVS) impression material in putty and regular body viscosities (Express XT, 3 M ESPE, St. Paul, MN, USA) to conduct a diagnostic mock‐up; an important tool for minimally invasive restorative clinical trials and preparations [46].

**FIGURE 4:**
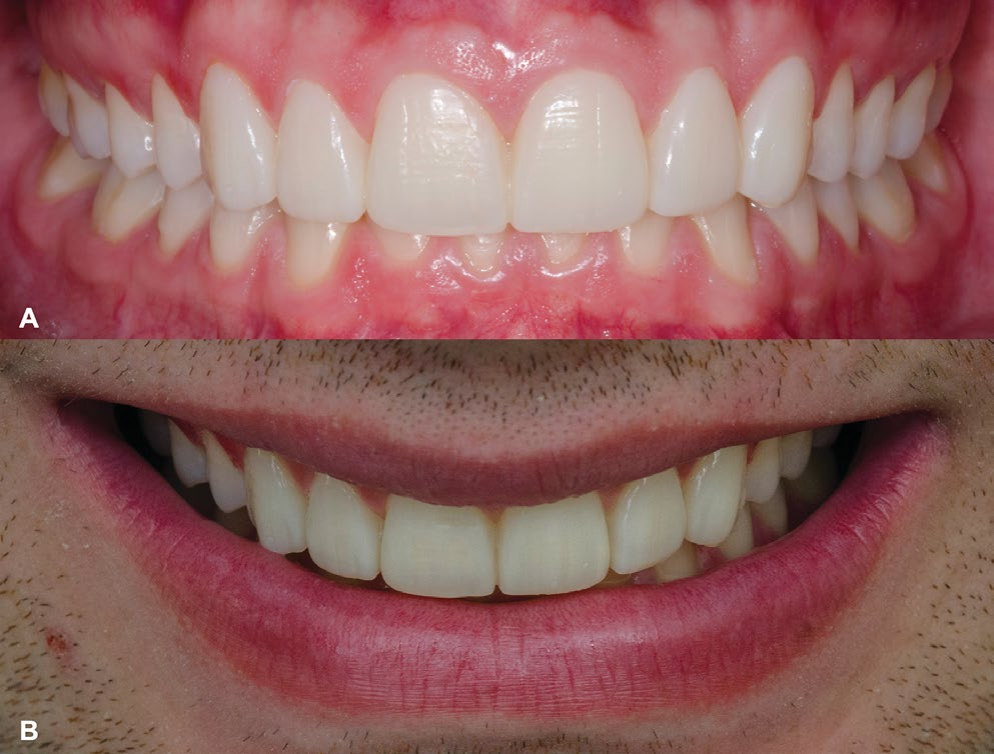
Views of the bis‐acrylic mock‐up. (A) Intraoral view with the patient in occlusion. (B) Frontal view of the new smile design.

The PVS silicone index covered the right maxillary premolar to the left one, and a “V”‐shapedcut was made at the cervical embrasures using a #15 scalpel blade to facilitate excess material drainage and removal. A self‐curing bis‐acrylic resin (Protemp 4, 3 M ESPE, Seefeld, Germany) in shade A1 was used to fabricate the mock‐up (Figure 4). During this phase, key esthetic and functional parameters—including tooth size, shape, proportion, position, surface texture, and occlusal relationship—were critically assessed by the clinicians and the patient. Both parties subsequently approved the proposed design.

**FIGURE 5:**
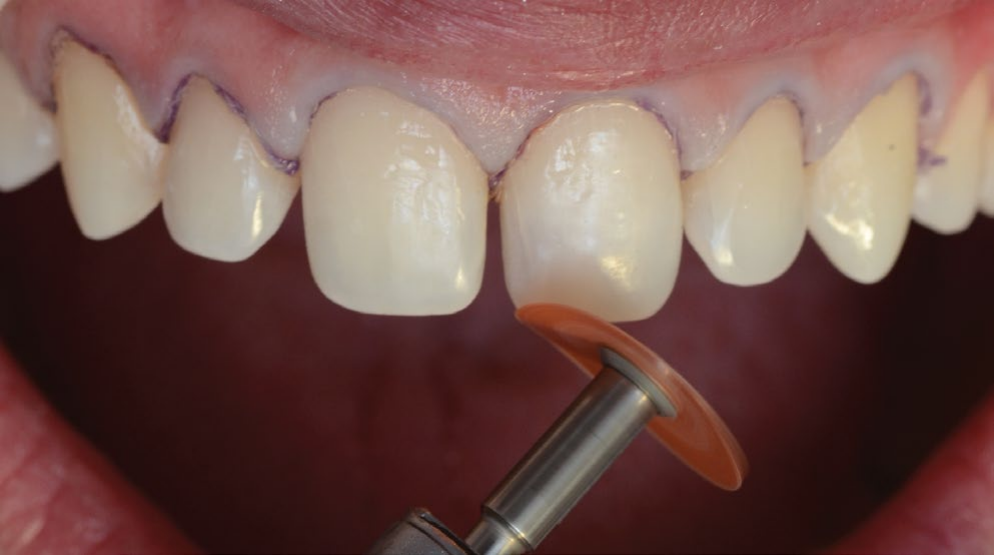
Dental preparations and adjustments with Sof‐Lex medium‐grit abrasive disc.

**FIGURE 6:**
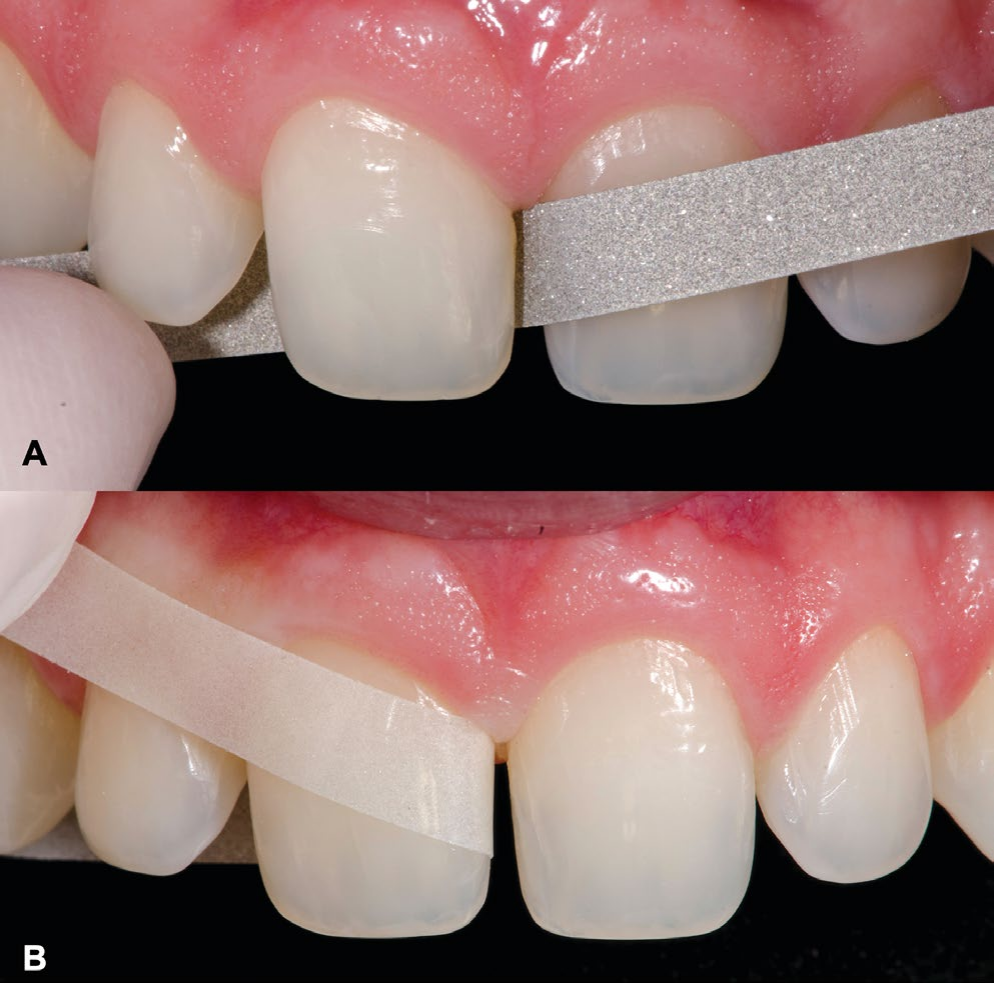
Interproximal adjustments. (A) With metallic strip. (B) With polyester strip.

Appropriate preparation geometry is essential to facilitate the insertion and accurate positioning of the ceramic restoration during the clinical trial and cementation process, thereby preventing undesirable contours, particularly in the dentogingival emergence profile. To ensure proper tooth preparation, a #0 knitted retraction cord (Ultrapak, Ultradent Products, South Jordan, UT, USA) was placed into the gingival sulcus of the involved teeth. Subsequently, a tapered round‐ended fine‐grained diamond bur (#2135FF; KG Sorensen, Barueri, SP, Brazil) was used to perform a slight wear (approximately 0.2 mm) of the enamel in the cervical region of the teeth—an area that may present aprismatic structure. Therefore, a minimally invasive knife‐edge finish line was created, not compromising the insertion axis of the ceramic laminates in a more oblique direction. In addition, minimal adjustments were performed, including the smoothing of edges and sharp angles on the buccal surface, and rounding of the labial‐incisal and palatal‐incisal angles. Medium‐grit abrasive discs (4931 M, Sof‐Lex Pop‐On, 3 M ESPE, St. Paul, MN, USA) mounted on a contra‐angle handpiece were used for this purpose (Figure 5). Additionally, minor interproximal adjustments were made using metallic and polyester strips to eliminate potential interferences between the adjacent teeth (Figure 6).

**FIGURE 7:**
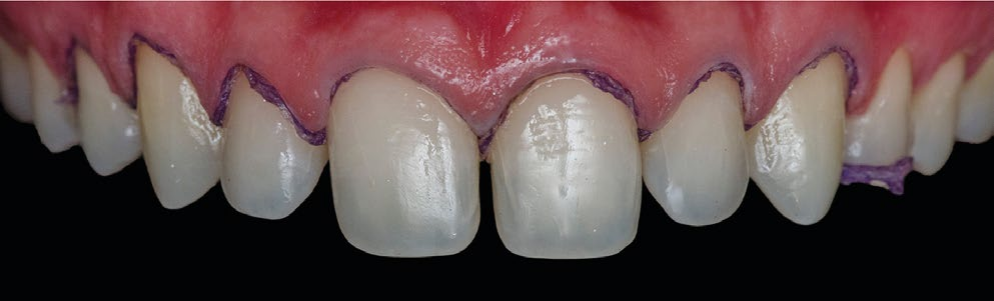
Teeth after preparation restricted to enamel surface.

**FIGURE 8:**
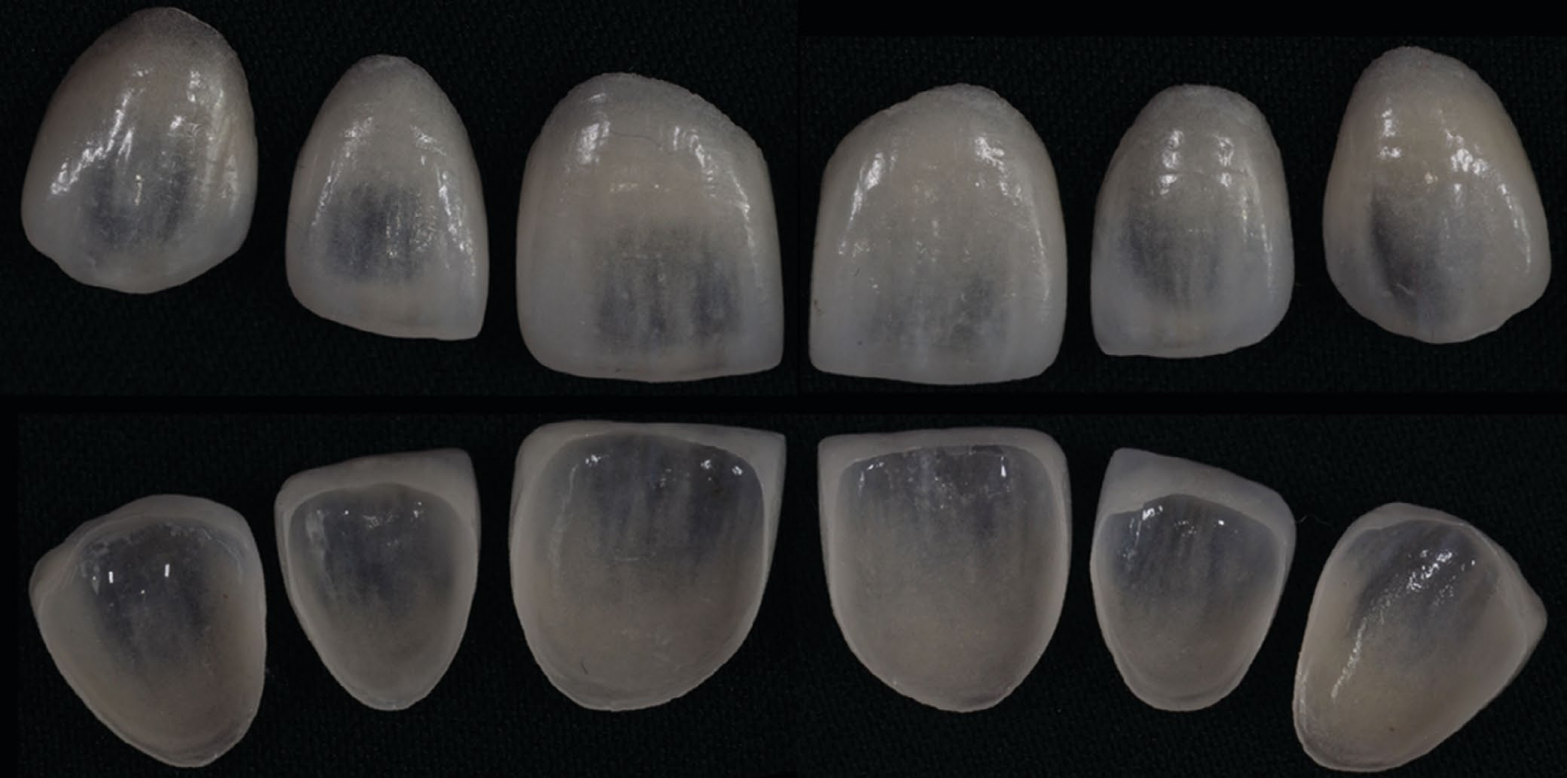
Feldspathic laminate veneers. Note the central region with a thin thickness and high translucency, allowing clear visualization of the black background used for contrast.

Following enamel‐limited tooth preparation (Figure 7), a maxillary final impression and an antagonist impression were obtained using a 2‐step technique with PVS impression material (putty‐reline technique + regular body; Express XT, 3 M ESPE, St. Paul, MN, USA). The impressions were disinfected and sent to the dental prosthetics laboratory for cast fabrication and the creation of refractory dies. Feldspathic porcelain laminates were fabricated by the dental technician André Luiz Vieira Ferraz (Figure 8) in shade A1 of the Vita Classical scale (VITA Zahnfabrik, Bad Säckingen, BW, Germany) using the Creation CC ceramic (Willi Geller International, Meiningen, Austria) by the powder/liquid layering technique. In Figure 8, it can be observed that minimum overlap was achieved after minimum or no palatal dental preparation. When made, these preparations were done with medium‐grit abrasive discs to obtain a featheredge finish line in the incisal‐palatal surface.

**FIGURE 9:**
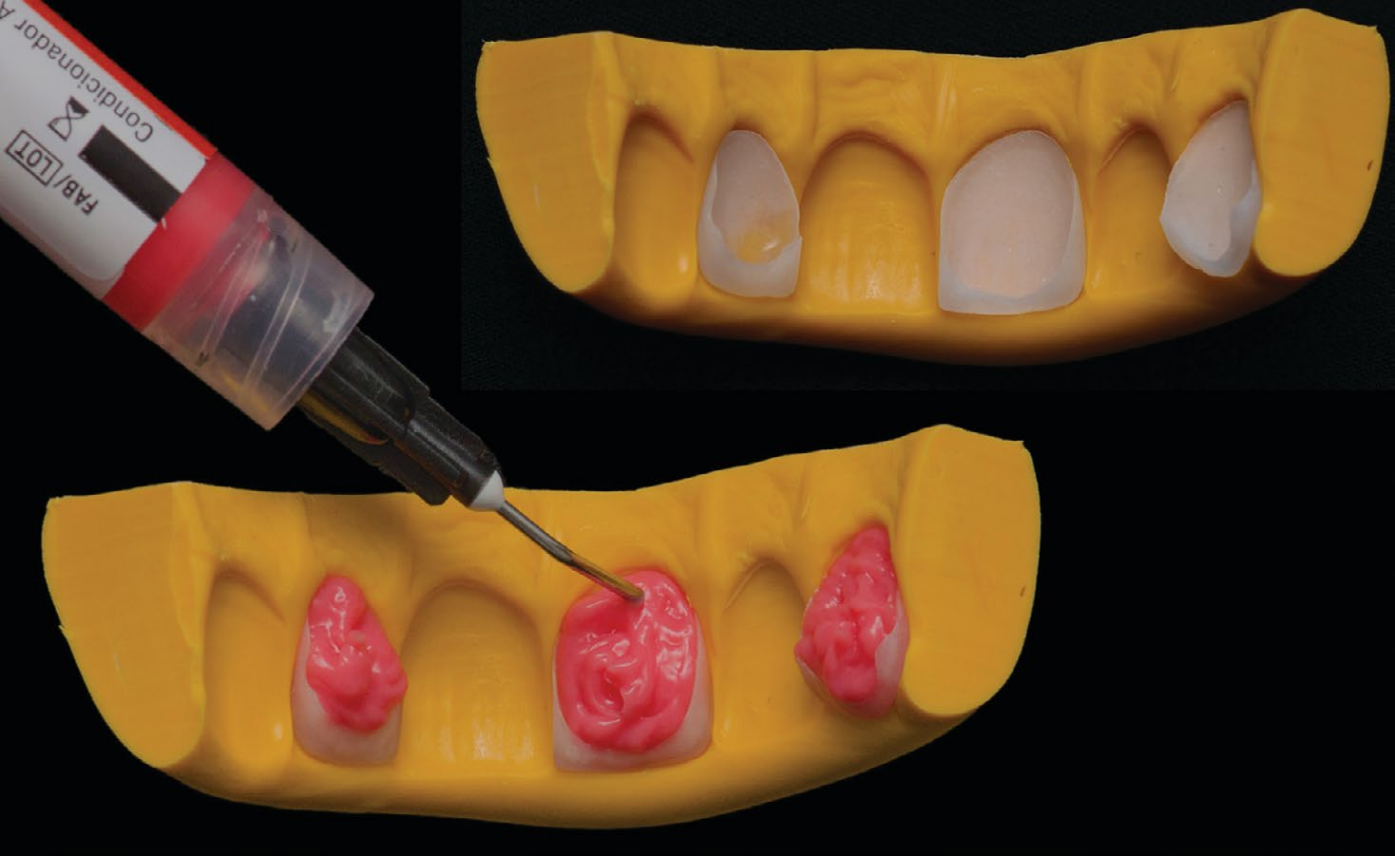
Etching the inner surface of veneers with hydrofluoric acid. The laminates were alternately positioned in the matrix to prevent the etching of undesired adjacent and glazed areas.

**FIGURE 10:**
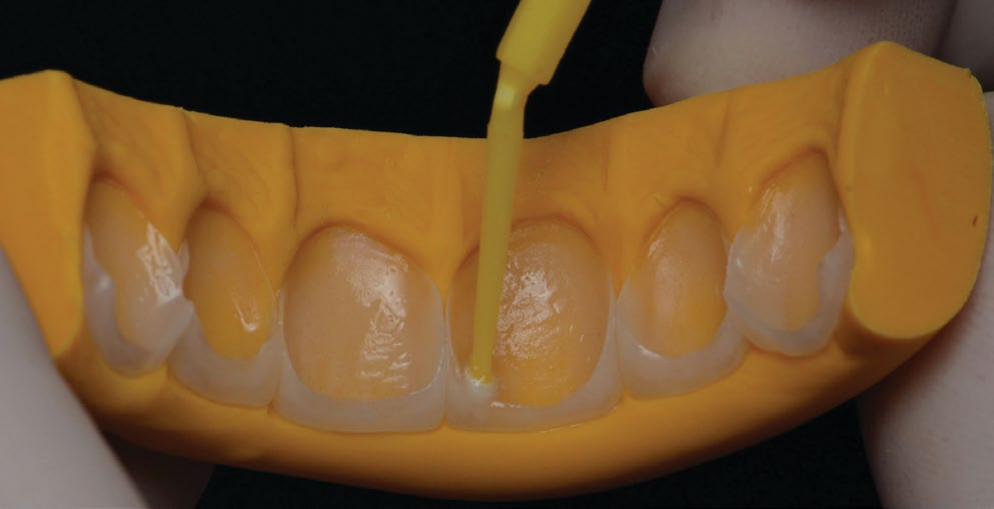
Silane application followed by unpolymerized adhesive.

Before the cementation procedures, a PVS matrix with a putty consistency (Express XT, 3 M ESPE, St. Paul, MN, USA) was fabricated to stabilize the ceramic veneers and facilitate their handling during the chairside surface treatments. This PVS matrix was not used to seat the veneers all‐in‐one during cementation. The inner surfaces of the ceramic restorations were etched with 10% hydrofluoric acid (Condac Porcelana, FGM, Joinville, SC, Brazil) (Figure 9) for 90 s, followed by thorough rinsing with running water and drying with compressed air. Subsequently, 37% phosphoric acid (Ultra‐Etch, Ultradent Products, South Jordan, UT, USA) was applied for 30 s using disposable microbrush tips to actively clean the vitreous dissolution, removing hexafluorosilicate residues generated during hydrofluoric acid etching [26]. After another round of rinsing and drying, silane (RelyX Ceramic Primer; 3 M ESPE, St Paul, MN, USA) was applied with friction for 60 s (Figure 10).

After placing a #000 knitted retraction cord (Ultrapak; Ultradent Products, South Jordan, UT, USA) around the prepared teeth to control gingival crevicular fluid, the operative field was isolated using the cotton roll isolation technique. According to Olegário et al. [47], this technique proved to be noninferior compared to rubber dam isolation in terms of restoration longevity. To ensure optimal results, six cotton rolls were placed in the maxillary labial sulcus from the left to the right molar area. A high‐vacuum saliva ejector was constantly maintained throughout the procedure. Gauze was also used in the palatine/lingual region and over the tongue to create a protective barrier against oropharyngeal humidity. The clinical procedure was carried out with six hands to maximize efficiency and to avoid contamination of the operative field.

**FIGURE 11:**
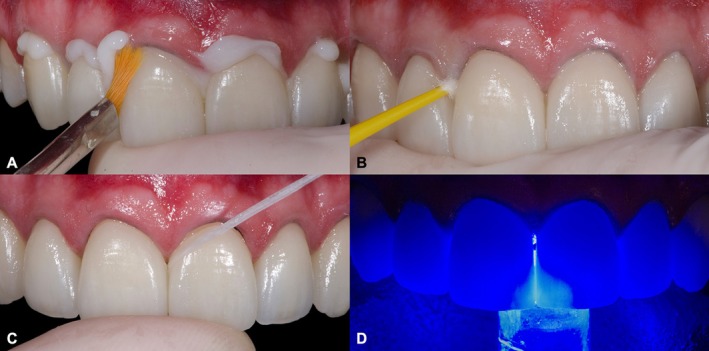
Cementation procedure and removal of the excesses. (A) With a flat brush. (B) With a disposable microbrush. (C) With dental floss. (D) Light‐curing.

Enamel etching was then performed using 37% phosphoric acid (Ultra‐Etch, Ultradent Products, South Jordan, UT, USA) for 30 s, followed by thorough rinsing and drying. Next, a conventional three‐step adhesive system (Scotchbond Multi‐Purpose, 3 M ESPE, St Paul, MN, USA) was applied according to the manufacturer's instructions. The light‐cured resin cement in shade TR (RelyX Veneer, 3 M ESPE, Seefeld, Bayern, Germany) was dispensed directly from its syringe on the inner surfaces of the ceramic laminate veneers, which were then seated onto the prepared teeth. After ensuring proper positioning of the veneers, excess cement was carefully removed using a synthetic hair flat brush, disposable microbrush tips, a dental probe, and dental floss. The polymerization of the cement was performed using an LED light‐curing unit with an irradiance of 1000 mW/cm^2^ (Ultrablue D‐2000; DMC, São Carlos, SP, Brazil), applying a 40‐s cycle to each surface of the teeth. During this procedure, the tip of the light‐curing unit touched the outer surfaces of the ceramic (Figure 11).

**FIGURE 12:**
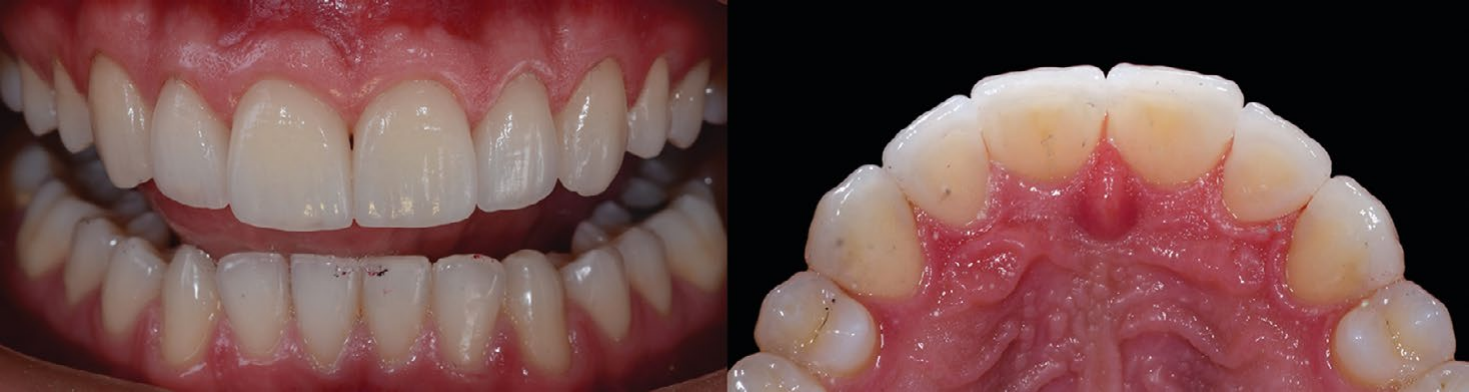
Verification of occlusal contacts.

Following the cementation of the veneers, the retraction cord was carefully removed, and the patient's occlusion was checked in maximum intercuspation. Additionally, anterior and canine guidance were also verified for eccentric jaw movements with posterior teeth disocclusion. To evaluate occlusal contacts (Figure 12) and ensure proper function, a 12‐μm‐thick carbon paper (AccuFilm, Parkell, Edgewood, NY, USA) was used. From the minimal palatal overlap planned since the mock‐up phase, it was possible to obtain the absence of dental contacts in the ceramic laminates in habitual occlusion.

**FIGURE 13:**
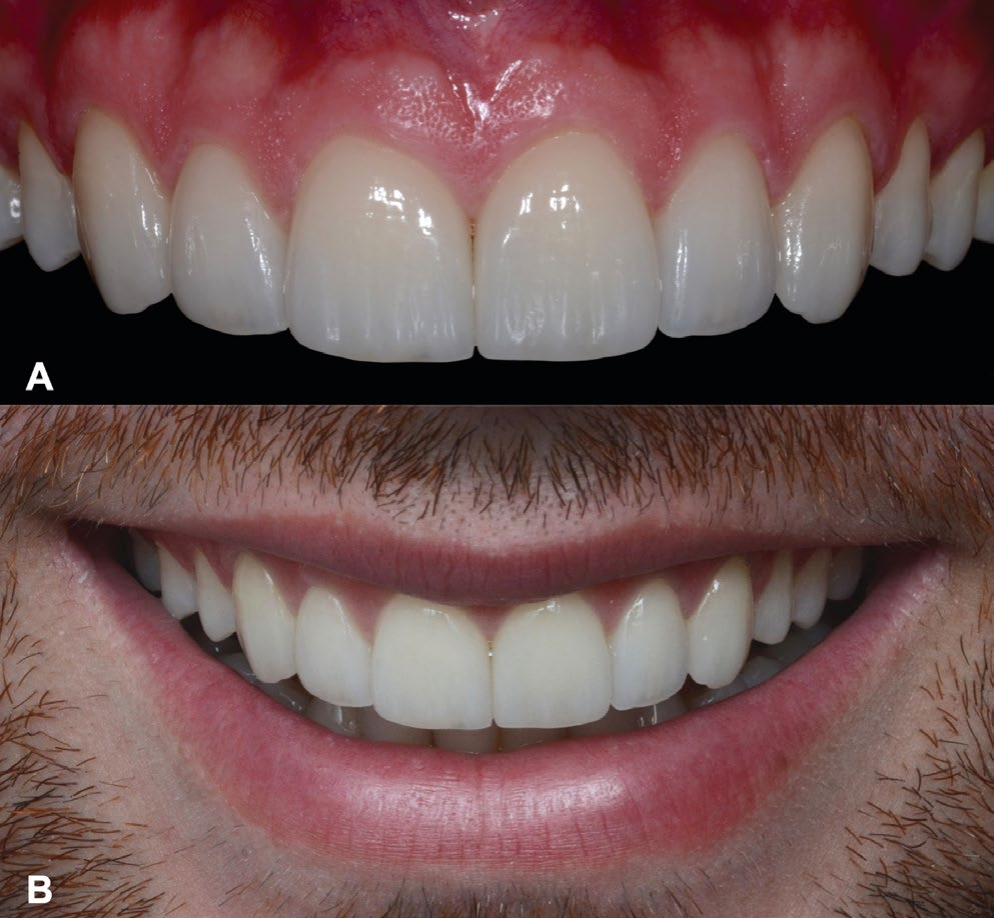
Final aspect of rehabilitation. (A) Intraoral frontal view. (B) Frontal view of the new smile.

**FIGURE 14:**
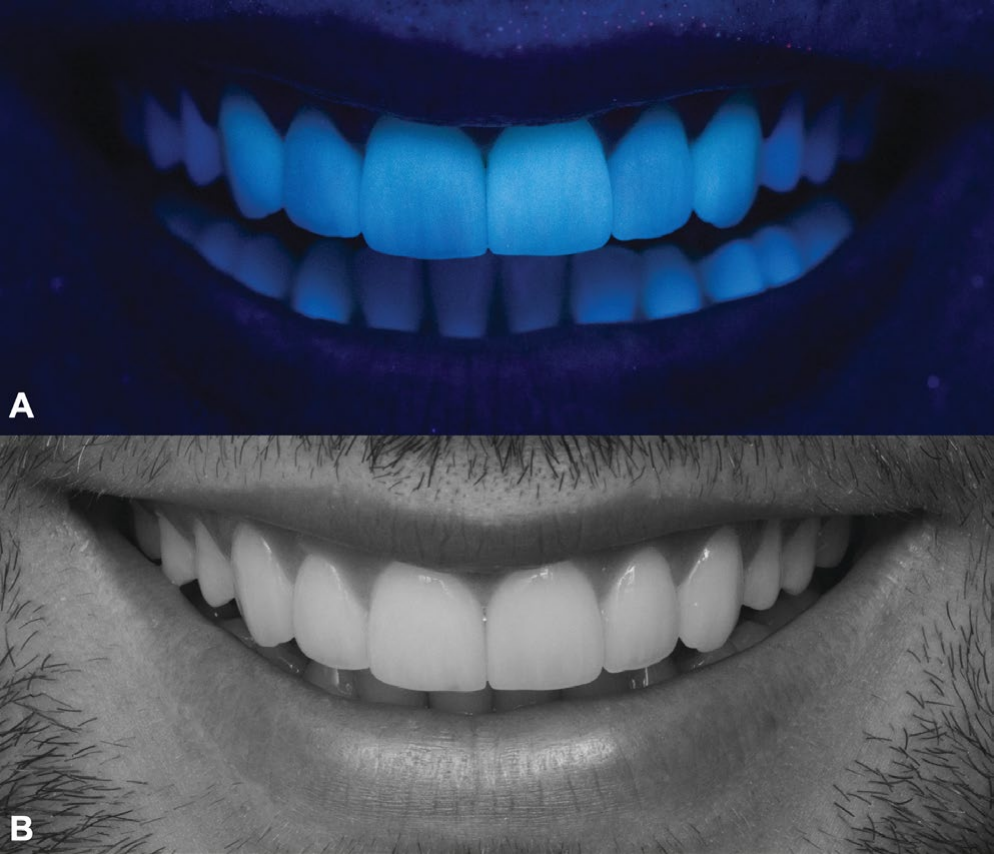
Analysis of optical properties. (A) Fluorescence. (B) Lightness.

The final esthetic outcome was assessed immediately after cementation (Figure 13), considering the integration of the restorations with the surrounding dentition and soft tissues. Furthermore, the fluorescence and value of the ceramic veneers were again examined to confirm their biomimetic characteristics and optical properties (Figure 14).

**FIGURE 15:**
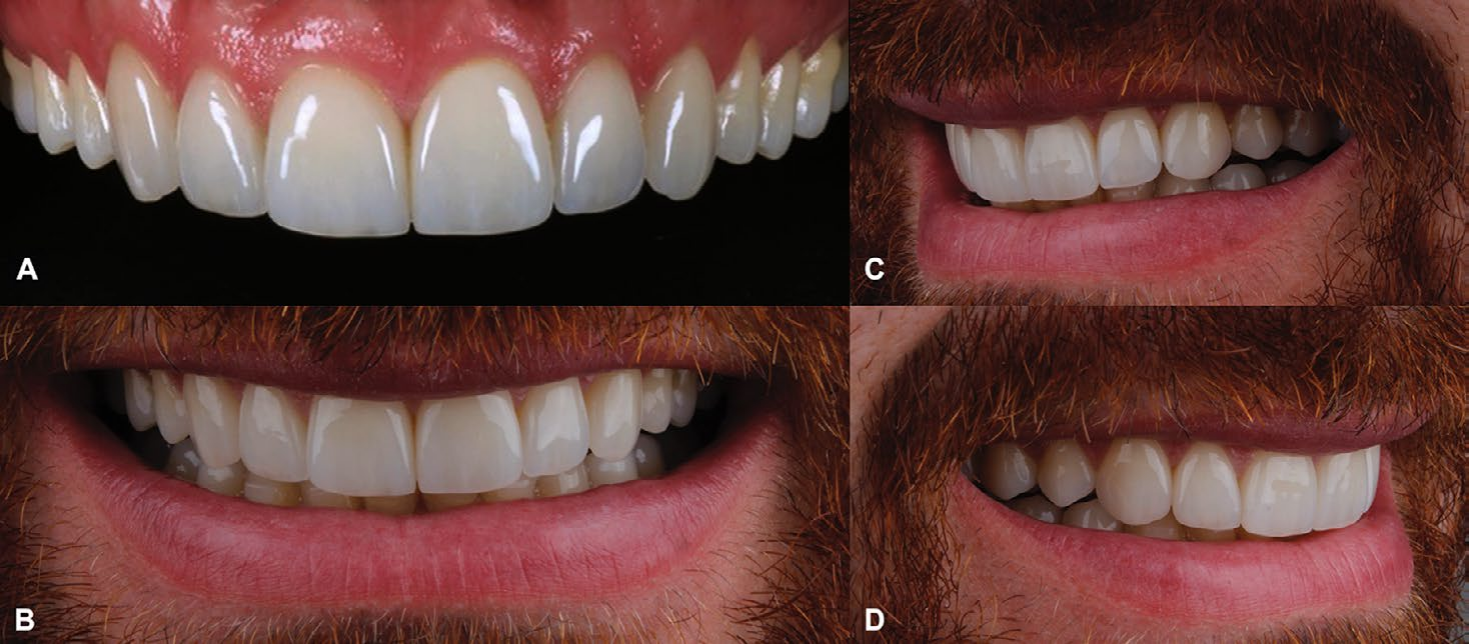
Rehabilitation at the last follow‐up visit. (A) Intraoral frontal aspect. (B) Frontal view of the smile. (C) Left lateral view of the smile. (D) Right lateral view of the smile.

**FIGURE 16:**
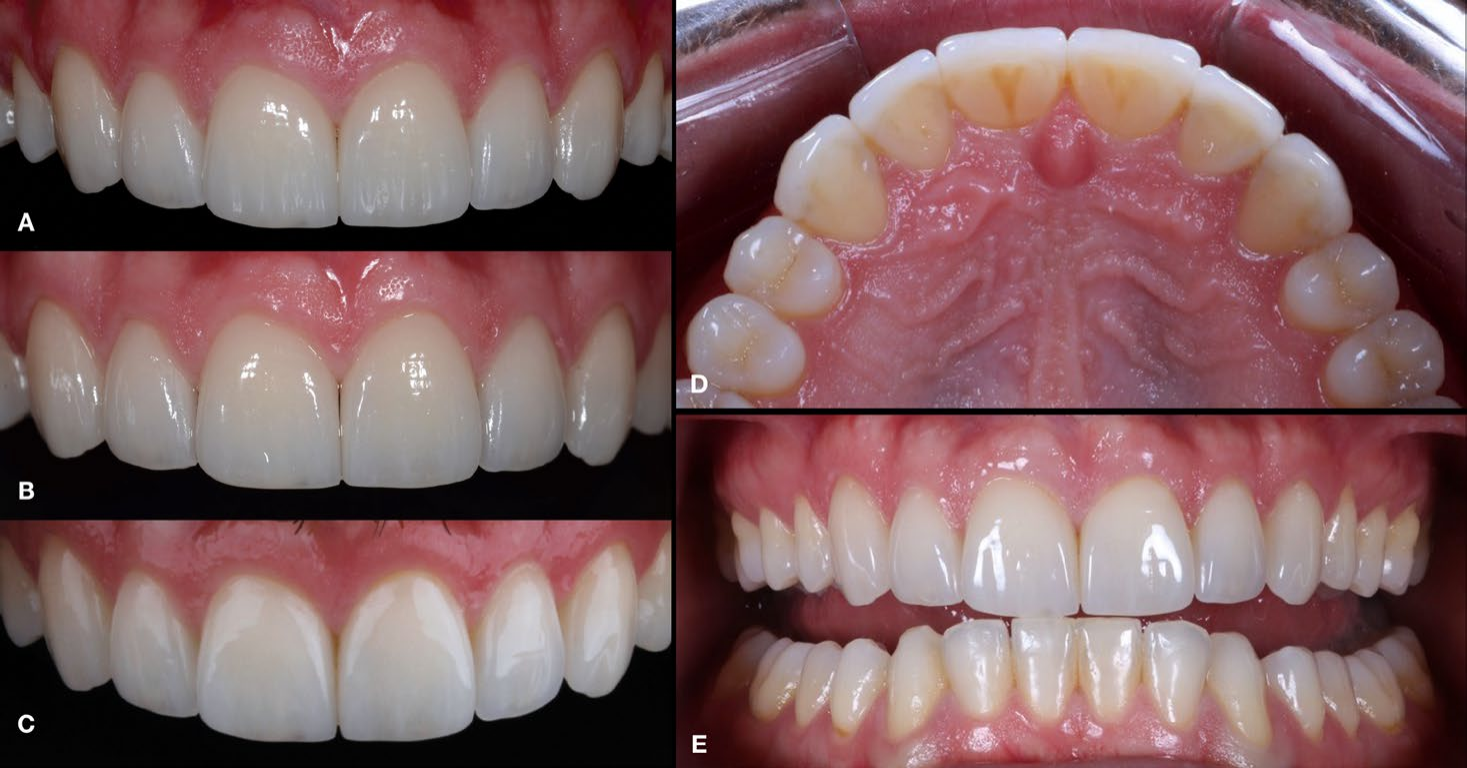
Follow‐up intraoral images. (A) Frontal view at baseline. (B) Frontal view at 3‐years follow‐up. (C) Frontal view at 12+ years follow‐up. (D) Palatal view at 12+ years follow‐up. (E) End to end teeth at 12+ years follow‐up.

The clinical follow‐up exceeded 12 years, during which no functional and/or esthetic complications were observed. A very subtle staining noted in some marginal areas did not negatively impact the overall esthetic outcome or the satisfaction of both the clinicians and the patient. This alteration in the clinical overview was primarily attributed to the natural apical migration of the gingival margin associated with aging, resulting in a predictable minimal cervical exposure. Concerning the biological behavior of the abutment teeth, no secondary caries, pulpitis, or pulp necrosis were observed. Moreover, the patient related no postoperative sensitivity in any period of the follow‐up. At the last appointment, the patient exhibited excellent outcomes and high satisfaction with the rehabilitation, highlighting the long‐term effectiveness of thin feldspathic porcelain laminates in minimally invasive treatments (Figures 15 and 16), concerning both restorative and periodontal principles.

## 3 Discussion

Minimally invasive dentistry is a treatment philosophy that prioritizes conservative techniques and biomimetic materials to preserve the natural dental structure while maintaining its integrity [1, 11, 17, 19, 20, 22, 46], together with preserving the health of the periodontal tissues [45]. In line with this approach, there has been a growing demand for esthetic dental treatments that offer immediate and long‐lasting results, with ceramic veneers emerging as one of the most sought‐after procedures in clinical practice over the past two decades.

For this clinical case, two treatment options were discussed with the patient to enhance their smile esthetics and close the diastemas. Among the available approaches, direct resin composite restorations and ceramic veneers were considered. After a thorough discussion regarding the advantages, disadvantages, and limitations of each option, feldspathic porcelain veneers were selected. This choice was primarily based on the outstanding optical properties of feldspathic porcelain, which allow for high levels of mimicry and long‐term esthetic stability [21, 35, 42, 43]. In a randomized split‐mouth clinical trial authors [31] observed that ceramic veneers on maxillary anterior teeth performed significantly better than composite indirect veneers after a decade in terms of survival rate and quality of the surviving restorations. Another relevant aspect in this case report is that the patient exhibited well‐aligned maxillary anterior teeth with a small, rounded morphology, a tendency toward a conical shape, and reduced volume, making them highly suitable for rehabilitation with ceramic veneers.

A key factor in the treatment decision was the minimal long‐term maintenance required by ceramic restorations compared with composite resins [31], as well as their superior esthetic performance. These benefits are attributed to the chemical composition, physical characteristics, and biological compatibility of ceramic materials, which contribute to enhanced durability and clinical longevity [8, 11].

The mock‐up serves as a crucial step in the treatment planning process, allowing patients to visualize the proposed rehabilitative outcome directly in their mouths before any irreversible procedures are performed. This phase provides patients with a realistic preview of the final result, enabling them to request modifications before the dental preparation stage and make an informed decision regarding the proposed treatment [46]. The patient expressed satisfaction with the in‐mouth mock‐up and consented to proceed with the proposed minimally invasive dental preparations. Consequently, selecting a ceramic material that closely replicates the optical properties of natural teeth, such as feldspathic porcelain, was essential [9, 11, 35, 41]. This material allows for the fabrication of thin veneers often below 0.5 mm in thickness on refractory dies, ensuring high translucency and exceptional esthetic integration [1]. Despite its esthetic advantages, feldspathic porcelain presents technical limitations, including low flexural strength (approximately 70–90 MPa) prior to adhesive cementation [5, 41]. Therefore, meticulous care is required throughout the manufacturing process, during handling at the try‐in stage, and particularly during the cementation procedure to prevent fractures or damage to the restorations.

The use of feldspathic porcelain for veneers is well supported, with studies indicating a high clinical longevity rate over 10 years of function [8, 9, 35, 40, 42]. In a long‐term clinical study [42], the survival and success rates of feldspathic veneers over a follow‐up period of up to 21 years were assessed, demonstrating that the durability of these restorations is highly dependent on their adhesion to the dental substrate. These findings were recently corroborated in a literature review [36] and in a systematic review and meta‐analysis [43] regarding the clinical performance of laminate veneers. The ideal adhesive conditions are achieved when (I) at least 50% of the bonding substrate is enamel and (II) 70% or more of the restoration margin remains in enamel [1, 18]. In the present case, minimizing tooth reduction prioritized enamel preservation. This approach not only maintains the health of hard and soft tissues [45], but also enhances the long‐term success of the veneers by ensuring optimal adhesion and resistance to failure [13, 42].

A comprehensive evaluation and meticulous treatment planning are essential in determining the most appropriate restorative material for each clinical situation. Advances in dental materials and adhesive techniques have enabled more conservative approaches, reducing the necessity for invasive procedures such as extensive tooth reduction for thick veneers or the fabrication of full crowns. Additionally, the cost–benefit ratio of minimally invasive restorative techniques should be carefully weighed against conventional restorative methods, particularly in cases where favorable tooth alignment allows for minimal preparations confined to the enamel surface.

## 4 Conclusions

Proper planning and the correct indication of dental ceramics contribute to the success of rehabilitative treatment with thin feldspathic porcelain laminates. The physical, chemical, and esthetic properties of this material, combined with conservative preparation restricted to enamel, favor the bonding results, longevity, and success rate of the treatment. The initial objective of achieving excellent esthetic results and patient satisfaction was accomplished, and follow‐up appointments will be conducted to evaluate the longevity of this rehabilitation, which has now been maintained for over 12 years without requiring repairs.

## Conflicts of Interest

The authors declare no conflicts of interest.

## Data Availability

The data that support the findings of this study are available from the corresponding author upon reasonable request.
